# The Costs of Scaling Up HIV Prevention for High Risk Groups: Lessons Learned from the *Avahan* Programme in India

**DOI:** 10.1371/journal.pone.0106582

**Published:** 2014-09-09

**Authors:** Sudhashree Chandrashekar, Lorna Guinness, Michael Pickles, Govindraj Y. Shetty, Michel Alary, Peter Vickerman, CHARME–1 Evaluation Group, Anna Vassall

**Affiliations:** 1 London School of Hygiene and Tropical Medicine, London, United Kingdom; 2 St John's Research Institute, Bangalore, India; 3 Imperial College, London, United Kingdom; 4 Karnataka Health Promotion Trust, Bangalore, India; 5 URESP, Centre de recherchedu CHU de Quebec, Quebec, Canada; 6 Département de médecine sociale et préventive, Université Laval, Québec, Canada; University of Washington, United States of America

## Abstract

**Objective:**

The study objective is to measure, analyse costs of scaling up HIV prevention for high-risk groups in India, in order to assist the design of future HIV prevention programmes in South Asia and beyond.

**Design:**

Prospective costing study.

**Methods:**

This study is one of the most comprehensive studies of the costs of HIV prevention for high-risk groups to date in both its scope and size. HIV prevention included outreach, sexually transmitted infections (STI) services, condom provision, expertise enhancement, community mobilisation and enabling environment activities. Economic costs were collected from 138 non-government organisations (NGOs) in 64 districts, four state level lead implementing partners (SLPs), and the national programme level (Bill and Melinda Gates Foundation (BMGF)) office over four years using a top down costing approach, presented in US$ 2011.

**Results:**

Mean total unit costs (2004–08) per person reached at least once a year and per monthly contact were US$ 235(56–1864) and US$ 82(12–969) respectively. 35% of the cost was incurred by NGOs, 30% at the state level SLP and 35% at the national programme level. The proportion of total costs by activity were 34% for expertise enhancement, 37% for programme management (including support and supervision), 22% for core HIV prevention activities (outreach and STI services) and 7% for community mobilisation and enabling environment activities. Total unit cost per person reached fell sharply as the programme expanded due to declining unit costs above the service level (from US$ 477 per person reached in 2004 to US$ 145 per person reached in 2008). At the service level also unit costs decreased slightly over time from US$ 68 to US$ 64 per person reached.

**Conclusions:**

Scaling up HIV prevention for high risk groups requires significant investment in expertise enhancement and programme administration. However, unit costs decreased with programme expansion in spite of an increase in the scope of activities.

## Introduction

UNAIDS estimates that annually US$ 22 billion is required to achieve universal access to HIV prevention, treatment, care and support services globally by year 2015 [1]. To ensure that global health targets for HIV can be achieved in the context of the economic crisis and the resulting flat-lining of development assistance for health, increased attention is focusing on the cost of HIV prevention. Evidence on the costs of HIV prevention can assist planners forecast the resource requirements, estimate the cost-effectiveness of services, and identify potential areas of efficiency improvement. Empirical estimates of HIV prevention costs based on data collection during programme scale-up, are particularly useful in assisting those working in HIV prevention predict how costs may vary with scale and programme evolution.

Previous studies of HIV prevention provide some indication of the costs of HIV prevention for high risk groups [2]–[13] however, these studies are limited in that they either estimate costs from a very small number of providers or over a short time period. *Avahan*, the India AIDS Initiative of the Bill & Melinda Gates Foundation, provides HIV prevention services to high-risk groups (HRGs) including female sex workers (FSWs) and high-risk men who have sex with men (HR-MSM) and transgender (TG) in six states in India. The goal of *Avahan* is to reduce HIV prevalence among HRGs and stabilize HIV infection rates among the general population [14]. Phase I of the *Avahan* programme aimed to scale up HIV prevention (2003–2008) and Phase II (2008–2013) to transition services to the National AIDS Control Organization (NACO), India. The scale of *Avahan* provides a unique opportunity to examine costs over a range of settings, over time and at different programmatic scales.

The study presented here is an update of previously published analysis of the costs of the first two years of *Avahan* scale up. This update includes additional data on the costs of HIV prevention for high risk groups at the national programmatic level. In addition, although all the *Avahan* interventions delivered a minimum package of interventions, as the programme evolved new elements were included such as the development of community mobilisation, building an enabling environment and vulnerability reduction. In summary, we present here a descriptive analysis of the costs of entire Phase I of the *Avahan* programme (2004–2008). In terms of both time frame and sample size this is the largest and the most comprehensive cost analysis of an HIV prevention programme globally to date.

## Methods

### Study Setting

We collected data from four of the six states served by *Avahan* (Karnataka, Tamil Nadu, Andhra Pradesh and Maharashtra). The two north-east states of Manipur and Nagaland are not included their epidemic is primarily driven by Injecting drug users. Details of HIV prevalence, incidence and the size of the key populations in each state are provided in Table 1. Our sampling was exhaustive: and within each state we costed all NGOs and supporting partners.

**Table 1:** Setting description.*

States	Total Population (2011 census)	HIV Prevalence (2011–12)	People living with HIV (2011–12)	Annual New HIV Infection (2011–12)	Estimated size of high risk population**		Avahan district high risk population estimates (2008)
	Millions	(%)			FSW	MSM	Total (FSW & MSM included)
Karnataka	61	0.5	209,000	9,024	119,600	24,900	69,256
Tamil Nadu	72	0.2	133,000	2,738	79,700	49,200	500,50
Andhra Pradesh	84	0.7	419,000	16,603	86,000	18,700	81,615
Maharashtra	112	0.4	316,000	5,893	245,800	101,000	53,874

*Source: NACO (2012). "Technical report India HIV estimates."

**includes both *Avahan* and Non-*Avahan* districts. Source: Avahan.

### Ethical Approval

Ethics approval was provided by the centre hospitalier affilie universitaire de Quebec, Canada; Health monitoring and screening committee (HMSC), India and Institutional ethical review board of St. John's Medical college and Hospital, St. John's Research Institute, Bangalore. Written informed consent was given by participants who participated in the study.

### Programme and services costed

The package of HIV prevention services costed includes outreach through peers, behavior change communication, condom distribution, clinical services for sexually transmitted infections (STIs), community mobilization, advocacy and enabling environment activities, and is outlined in detail in Table S1. Peer educators provide services to about 25–50 people each, sharing prevention information, distributing supplies (condoms and lubricants) and providing referral for STI management. Referral clinics followed standard protocols for STI management^15^. Community mobilization, advocacy and enabling environment activities varied across the sites and included the formation of self-help groups, various drop-in center events, skills training, legal literacy workshops, police and stakeholder sensitization, crisis response teams and access to social entitlements [15]. Anti-retroviral therapy was not included in costs as it was not part of the package. There was active referral of individuals for HIV testing and positive key populations were referred to government anti-retroviral treatment (ART) centers for care and support.

HIV prevention across all four states was guided by a common minimum programme [15]. These included a set of implementation standards for technical and managerial areas, project milestones, a common management framework, and a common set of indicators. Beyond this there was flexibility to adapt services based on local context.

In the four study states, *Avahan* was implemented in 64 of the total 120 districts in the four southern states by 138 NGOs, supported by six state level lead Partners (SLPs) contracted by the Bill and Melinda Gates foundation, which also had a national level office at Delhi. In Karnataka and Tamil Nadu there was one SLP each, while NGOs in the states of Maharashtra and Andhra Pradesh were supported by two SLPs each. In 2003–4 SLPs identified NGOs for sub-granting, worked with these NGOS to conducted state wide mapping of high risk populations and co-ordinated closely with state AIDS control societies (SACS) to avoid the duplication of activities. This mapping was conducted by *Avahan* NGO partners or through contracted technical assistance agencies. All partners used variations of a non-mathematical method that involved a combination of geographic and social mapping combined with the iterative intensive use of Delphi techniques with different key informants. Most of the partners repeated the indirect non-mathematical size estimation exercise on an annual basis [16].

Thereafter SLPs provided technical assistance to develop key programme strategies, developed communication materials, enhanced the expertise of NGO staff, provided supportive supervision and consolidated the programme outputs through computerised management information system (CMIS), and supported the purchase and distribution of commodities. In their grant management role SLPs reviewed programme implementation and financial reporting of the NGOs.

At the national level, *Avahan* foundation office developed over-arching programme strategies and organised annual partners meetings to share lessons learned and co-ordinate with the Indian authorities. They also co-ordinated with the agency sub-contracted for setting up and maintaining the centralised management information system, provided financial oversight and monitored programme evaluation. International NGOs and academic institutions were contracted in by the national office to provide pan-*Avahan* technical support. Technical assistance was primarily focused on enhancing the expertise to deliver STI services, provide advocacy, inter-personal communication and community mobilisation.

All NGOs were registered and operating prior to *Avahan* in sectors like education and poverty alleviation. A few NGOs had previous experience in HIV prevention. In some districts, *Avahan* funded NGOs were the sole provider of HIV prevention services in the district while in other districts provision was shared with NGOs funded by the National AIDS control organisation (NACO) with distinct catchment areas.

### Cost data collection

We collected cost data for each year of Phase 1 (2004–8) prospectively from a provider perspective. Further details can be found in our initial two year study, but are outlined in summary here [17]. We included all costs of HIV prevention for female sex workers (FSWs) and high risk men who have sex with men and transgendered persons (HR-MSM/TG) in four states. Excluded from the analysis were costs associated with evaluation and research-related activities, support to non-*Avahan* districts, condom social marketing, a separate male STI service delivery program, and client intervention and a trucker focused intervention. These costs were excluded as they are not a part of core activities of NGO's interventions for the targeted high risk groups.

The costing approach used was primarily top-down – allocating out expenditures and economic costs to districts then NGOs and then to activities [17]. This was aided with comprehensive access to expenditure data, time sheets, the bottom up data collection of economic costs not included in financial records, and interviews with staff. Expenses prior to the first person being reached by the programme were treated as start-up costs and annuitized: costs are therefore reported from 2004.

Costs were also disaggregated and categorized by activities and input type. A full list of the inputs included are presented in Table S2 (and activities in Table S1). In 23 of the districts we conducted a detailed cost analysis of 37 NGOs including field visits and time sheets to estimate the share of costs allocated to different NGO sub-activities (outreach, community mobilization etc.). In non-detailed districts costs were estimated using expenditure records, commodity distribution records, financial records and narrative reports (for example to estimate the number of volunteer peer educators) submitted to the SLP. In some districts NGOs were replaced by new NGOs due to non-compliance of financial procedures. This led to temporary gap in service till another NGO was identified to take over the interventions. In this case, we included expenditures of both the NGOs for that district.

*Avahan* programme management costs (at the national level) were allocated among all grantees according to size of the expenditures for grant in year of analysis; and then from SLP's to districts and NGOs based on estimated population size. This allocation criteria was selected following extensive discussion with programme managers; who reported that estimated population size was the criteria they used to apportion their efforts. A more sophisticated method was used for allocating SLP level expenses to each NGO, first allocating out specific expenditures that could be clearly tracked to particular NGOs, then, for remaining expenditures, allocating to activities on the basis of SLP staff interviews, and thereafter allocating costs either equally or by persons reached to each NGO, depending on the activity. This procedure was determined following extensive interviews and discussions with SLP staff on how they spent their time and resource for different programme activities.

We report economic costs. Financial costs represent expenditure on goods and services purchased. Economic costs include items for which there were no financial transactions, for example volunteer time and/or donated goods. These goods were valued using market prices. Where peer educators were volunteers, we used the NACO peer honorarium as the market price. Economic capital costs were annualised using a discount rate of 3%.

Unit costs were estimated using output indicators obtained from the Computerised Management Information system (CMIS) [18]. We report two types of unit costs: cost per person reached at least once a year and per monthly contact made. Per person reached is defined as number of people reached at least once in the year being costed. Monthly contact made is defined the number of individuals contacted in any one month, summed over the year. If an individual is contacted more than once in a month, then this is still counted as one contact. Counting all contacts was not possible as this data was not reported to the CMIS. While this measure underestimates the true number of contacts, estimates from programme and NGO manager interviews suggest that less than 5% of high risk group persons contacted are likely to have been more than once a month in any particular month.

We followed a participatory approach during data collection. We utilised the common platforms during *Avahan* partners meetings and SLP meetings to brief participants about the study methods, and receive feedback on preliminary results. Other key participants included NGO staff and SLP local partners. Extensive work was carried out to ensure the full involvement of participants in this study: and this was particularly helpful in terms of validating our results. Data were entered and analysed using Microsoft Excel. All costs are presented in using US dollars 2011 (www.data.worldbank.org/indicator). The dataset on costs presented here is available from the corresponding author on request.

Ethics approval was provided by the centre hospitalier affilie universitaire de Quebec, Canada, Health monitoring and screening committee (HMSC), India and St. John's Research Institute, Bangalore. Written informed consent was given by all participants who participated in the study.

## Results

The total economic cost of the Phase 1 scale-up of *Avahan* was US$ 102,057,077 of which US$ 35,890,683 was spent at the service level (NGO level)(35%) and US$ 66,166,394 (65%) at above service level (**Table 2**). The total cost of the programme increased over the years, particularly between the first and second years of scale-up. After year 1, proportion of costs above the service level remained relatively constant, whereas both the proportional and total cost incurred at NGO level increased (from 24% in 2004 to 44% in 2008). Programme management (28%), advocacy (24%), interpersonal communication support (15%) community mobilisation (11%) and support to STI services (10%) were the largest costs items at the national level (**Table 2**). Proportional costs between these different areas of activities remained relatively stable over the period.

**Table 2:** Total economic costs by organizational level and activity 2004 to 2008 (US$ 2011).

Activity	Economic costs (3%)									
	2004-05.	%	2005-06.	%	2006-07	%	2007-08	%	Total	%
**Programme Level**										
Programme management general	2,115,355	50	2,900,945	26	2,515,022	25	2,255,430	23	9,786,752	28
Support to inter-personal communication	558,398	13	2,056,492	18	1,427,460	14	1,410,729	15	5,453,080	15
Support to policy advocacy	0	-	1,191,432	11	1,748,137	17	1,437,190	15	4,376,759	12
Support to community mobilization	340,178	8	1,196,945	11	1,289,718	13	1,110,205	12	3,937,045	11
Support STI services	316,088	8	1,085,958	10	1,024,025	10	1,132,423	12	3,558,494	10
Support to advocacy with societal leaders	359,741	9	1,118,739	10	650,751	6	728,250	8	2,857,481	8
Support to programme management of expertise enhancement partner	334,743	8	988,674	9	846,411	8	822,101	9	2,991,928	8
Support to media advocacy	41,895	1	406,856	4	454,182	4	436,718	5	1,339,651	4
Support to strengthening HIV positive networks	138,900	3	274,641	2	305,042	3	290,985	3	1,009,568	3
**Sub-total (35% of total 2004-8)**	**4,205,299**	**100**	**11,220,682**	**100**	**10,260,748**	**100**	**9,624,030**	**100**	**35,310,758**	**100**
**State Level lead implementing Partner**										
Programme management	1,083,383	31	3,717,518	46	4,380,744	47	4,967,782	50	14,149,427	46
Expertise enhancement including technical assistance and training	834,561	24	1,543,047	19	1,762,620	19	1,759,232	18	5,899,460	19
Support and supervision	645,428	19	1,441,424	18	1,446,276	15	1,947,745	20	5,480,873	18
Management information	594,063	17	484,377	6	647,274	7	324,524	3	2,050,238	7
Information Education and communication	217,165	6	670,680	8	564,348	6	335,358	3	1,787,551	6
Community mobilization and enabling environment	101,946	3	251,942	3	604,009	6	530,191	5	1,488,087	5
**Sub –total (30% of total 2004-8)**	**3,476,546**	**100**	**8,108,987**	**100**	**9,405,271**	**100**	**9,864,832**	**100**	**30,855,636**	**100**
**Service Level (NGO** * **)**										
STI Services	635,724	27	2,636,754	36	4,011,918	36	5,239,684	35	12,524,080	35
Outreach, condom promotion behavior change and communication	643,064	27	1,598,516	22	2,536,109	23	3,456,964	23	8,234,653	23
Programme management	670,122	28	1,433,496	20	1,675,516	15	2,641,394	17	6,420,528	18
Community Mobilization and enabling environment	237,263	10	1,050,830	15	1,976,768	18	2,498,680	16	5,763,540	16
Expertise enhancement including technical assistance and training	207,783	9	520,819	7	907,921	8	1,311,359	9	2,947,882	8
**Sub-total (35% of total 2004-8)**	**2,393,957**	**100**	**7,240,415**	**100**	**11,108,232**	**100**	**15,148,080**	**100**	**35,890,683**	**100**
**Grand total**	**10,075,802**		**26,570,083**		**30,774,250**		**34,636,943**		**102,057,077**	

* 2 NGOs excluded.

**Figure 1:**
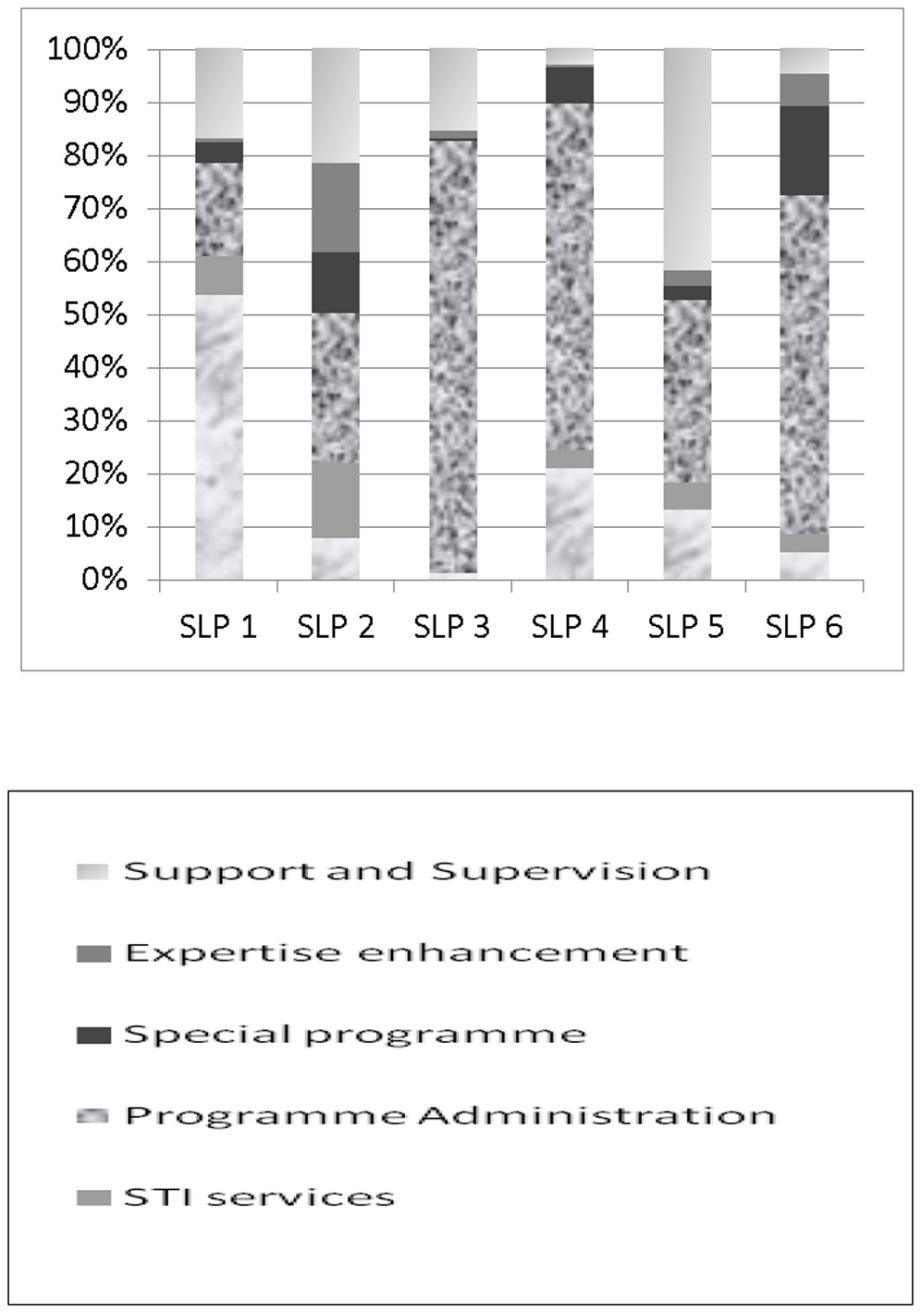
Phase 1 (2004-8) SLP economic costs by activity (%).

Programme management (46%), support and supervision (18%) and expertise enhancement (19%) were the activities that incurred costs by SLPs (**Table 2**). The proportional costs of both programme management and support to community mobilisation increased over the years, whereas the proportional costs of management information and information, education and communication fell. Different SLPs had markedly different cost patterns (**Figure 1**) – with some SLPs having higher costs for programme administration and others for expertise enhancement.

At the service level (**Table 2**), the proportional cost incurred for programme management fell over the years (from 28% to 17%), although the total programme management cost increased. Outreach costs and expertise enhancement stay constant as a proportion of cost throughout the period (around 23% and 8% respectively), although the total costs of both these activities increased. Both the proportional and total costs for areas such as STI services, community mobilisation increased (from 27% to 35%, and 10% to 16% respectively) – related to the expansion of clinics and clinical services including syphilis testing and TB verbal screening, the addition of new community mobilisation initiatives including organizational capacity building, advocacy and the expansion of enabling environment activities.

**Figure S1** shows the distribution of costs for all 64 districts across the period. There are substantial differences between districts on the proportion spent for each activity, particularly for STI services. Additional analysis of start-up costs (not shown) found that the mean start-up time for the 23 detailed costing districts was 4.8 months (ranging from 1 to 8 months). Start -up costs ranged from 2 to 8% of the total costs across all NGOs.

**Table 3** presents the cost profile by input for the NGOS and SLPs (this breakdown is not available for programme level costs). The largest area of cost was incurred by personnel at both levels (around 40%), with supplies costs being a major area of NGO level expenditures (27%). The total cost of supplies and commodities increased substantially over time at the NGO level. However proportional input costs at both levels remained fairly constant over the years. **Figure S2** shows the variation in cost profiles of NGOs and SLPS by district. The items that show the highest degree of proportional variation are commodities and supplies, buildings and indirect expenses, and travel costs.

**Table 3:** SLP and NGO economic costs by organisational level and input 2004 to 2008 (US$ 2011).

SLP LEVEL										
	2004-05	%	2005-06	%	2006-07	%	2007-08	%	Total	%
**INPUT**										
Personnel	1,524,017	44	3,469,329	43	3,491,036	37	3,940,084	40	12,424,467	40
Commodities and supplies	328,734	9	968,044	12	1,186,760	13	1,174,455	12	3,657,992	12
Trainings	315,144	9	639,004	8	1,301,959	14	1,068,110	11	3,324,217	11
Capital cost	335,558	10	740,897	9	772,088	8	862,258	9	2,710,800	9
Building operating & maintenance	134,439	4	715,514	9	912,959	10	866,836	9	2,629,748	9
Travel	272,166	8	608,406	8	576,317	6	826,892	8	2,283,781	7
Monitoring & Evaluation	493,897	14	603,413	7	757,733	8	445,537	5	2,300,579	7
Indirect Expenses	72,592	2	364,381	4	406,418	4	680,660	7	1,524,051	5
**Grand Total**	**3,476,546**	**100**	**8,108,987**	**100**	**9,405,271**	**100**	**9,864,832**	**100**	**30,855,636**	**100**

NGO LEVEL										
INPUT	2004-05	%	2005-06	%	2006-07	%	2007-08	%	Total	%
Personnel	1,031,110	43	3,388,765	47	4,753,485	43	6,601,159	44	15,774,520	44
Commodities and supplies	448,652	19	1,799,082	25	3,405,363	31	4,104,490	27	9,757,587	27
Capital cost	349,802	15	805,142	11	1,029,404	9	1,293,651	9	3,477,999	10
Travel	154,713	6	476,113	7	726,209	7	1,015,032	7	2,372,066	7
Building operating & maintenance	168,664	7	402,760	6	537,197	5	1,155,525	8	2,264,145	6
Trainings	108,229	5	238,146	3	404,776	4	673,152	4	1,424,302	4
Monitoring & Evaluation	124,487	5	95,659	1	93,375	1	158,257	1	471,777	1
Indirect Expenses	8,300	0	34,748	0	158,422	1	146,816	1	348,286	1
**Grand Total**	**2,393,957**	**100**	**7,240,415**	**100**	**11,108,232**	**100**	**15,148,080**	**100**	**35,890,683**	**100**

Programme outputs and unit costs are presented in **Table 4** **.** By year 4 the *Avahan* programme in the four districts was reaching over 300,000 target population members at least once a year, and the number of monthly contacts was over 2 million. The mean total unit costs (2004-08) per person reached at least once a year and per monthly contact were US$ US$ 235(56-1864) and US$ 82(12-969) respectively. NGO level unit costs per person reached fell slightly over the period at the service level (US$ 68 to US$ 64), but total unit cost per person reached fell more substantially as the programme expanded due to sharply decreasing unit costs at the above service level (from US$ 477 in 2004 to US$ 145 in 2008). While the variation of unit cost per person reached at the service level narrowed over time, this narrowing was more distinct at the above service level. Median unit costs were considerably lower than the mean costs with a smaller inter quartile range values as a few very high values skew the distribution of unit costs.

**Table 4:** Outputs and unit costs by service level 2004-08, US$ 2011.

Output Indicators	2004-05	2005-06	2006-7	2007-8
Persons reached at least once a year*	46,825	151,914	225,585	300,716
Monthly contacts**	179,343	640,770	1,256,743	2,052,218
Intensity of Contacts***	3.8	4.2	5.6	6.8

Unit cost in(US $2011)	2004-05	2005-06	2006-07	2007-08	2004-08
**Service level**					
**Per person reached**					
Mean (SD)	68 (89)	67 (54)	61 (44)	64 (57)	64 (47)
Range	11-425	19-412	10-285	13-459	13-268
Median (inter quartile Range)	38 (34)	54 (43)	53 (40)	47 (35)	51 (36)
**Per monthly contact**					
Mean (SD)	28 (42)	25 (39)	14 (16)	11 (14)	17 (20)
Range	3-224	3-249	3-116	2-124	3-131
Median (inter quartile Range)	10 (29)	13 (15)	8 (7)	7 (5)	10 (17)
**Above service level**					
**Per person reached**					
Mean (SD)	408 (960)	226 (376)	116 (141)	81 (65)	171 (232)
Range	45-5413	35-2923	13-1360	4-406	33-1650
Median (inter quartile Range)	116 (210)	120 (76)	90 (60)	65 (44)	100 (83)
**Per monthly contact**					
Mean (SD)	222 (534)	89 (219)	29 (47)	17 (28)	64 (122)
Range	5-2708	6-1720	2-380	1-244	6-838
Median (inter quartile Range)	34 (177)	33 (57)	14 (19)	9 (9)	30 (45)
**Total unit costs**					
**Per person reached**					
Mean (SD)	477 (1031)	292 (399)	178 (167)	145 (112)	235 (258)
Range	67-5838	56-3078	38-1506	41-866	56-1864
Median (inter quartile Range)	159 (260)	181 (103)	139 (94)	113 (84)	156 (95)
**Per monthly contact**					
Mean (SD)	251 (570)	112 (243)	42 (57)	27 (39)	82 (136)
Range	8-2932	3-1859	7-421	3-332	12-969
Median (inter quartile Range)	52 (215)	47 (67)	23 (26)	16 (16)	44 (61)

*Persons reached includes individuals contacted at least once a year.

** Sums the number of persons contacted at least once each month for all months in the year.

***Intensity is the number of monthly contacts per person reached.

## Discussion

This descriptive cost analysis of the *Avahan* programme presents the most comprehensive study to data on the changing cost structure of HIV prevention to high risk group during scale-up, updating our previous two year costing study [17]. Our previous analysis found a median cost per registered key population of US$76 compared to our new estimate of US$156. However, this increase in cost is primarily due to the fact that we are now able to take into account the full above service level cost that was not included earlier; an overall unit costs per person reached declined during scale-up. In the initial years of scale-up a high proportion of cost is incurred above the service level and then, as the programme expands and matures, a greater share of funds is channelled to NGOs. The proportion of cost for different activities also evolves during programme scale-up, with the initial focus being on outreach; and thereafter an expansion of funding for STI and community mobilisation. However, service level unit costs stay relatively constant over time, possibly as cost increases related to the expansion of programme scope are balanced out with cost reductions from scale.

Our mean service level unit cost estimates (US$64) are higher than those from other studies US$ 32(22-57) [7], US$ 57 [11], US$ 19(10-51) [13] and US$ 31(34-51) [19]. The range of services provided by *Avahan* is broader than that in the intervention package costed in many of these other studies^21^. In addition, cost differences may be due to the different estimation methods used. We primarily use a top down method, which may be less precise in terms of disaggregating costs at the activity level, but may better capture total cost. Previous studies only focus on small sample of NGOs who agreed to participate, and thus may suffer from selection bias. Finally, some of the previous studies did not include STI services costs which were referred to government hospitals or private providers.

One of the new and central findings of this update is that the greatest proportion of costs is incurred above the service level. Assessing the appropriate level of above service costs is complex. Aside from programme management, much of the above service cost was used to enhance the expertise of service providers. India has a robust NGO sector: and, in this sense, the degree of support required to provide quality services quickly may be less than elsewhere. The scale-up of services was also rapid, reaching full coverage of FSWs in most sites by the second year of the programme: and slower scale up may require less support. The programme then continued to expand to include coverage of HR-MSM/TG during 3^rd^ and 4^th^ year. However, although total above service level costs did not escalate during scale-up, the variation between expenditures by different SLPs suggests that there may be room for efficiency gain – and further qualitative and quantitative work is required to better understand the causes of this variation and how different patterns of above service level costs may relate to NGO performance. At the very least, our findings highlight that other countries expanding HIV prevention to high risk groups need to critically examine and plan their above service support activities with the same level of scrutiny as they monitor costs of those directly providing services.

Our updated results add further support to our previous evidence on the economies of scale of HIV prevention to key populations [17]. Economies of scale are driven by the extent to which areas of costs remain constant (or fixed) as the level of service increases. Previous studies have focused primarily on service level costs and suggest that costs are lower for larger NGOs than smaller ones. Our descriptive analysis presented here also suggests further economies of scale may be derived from fixed above service level costs. The dataset presented here will also be analysed econometrically to assess the specific extent of any scale effect at the NGO level. However, the fixed nature of the above service costs incurred indicates that economies of scale at a programmatic level may be substantially higher than our previous estimates considering service level costs alone.

We also find slight decline in unit costs at the service level as the programme scales up in spite of increase in the scope of services and difficulty of reaching more dispersed groups [20]. As *Avahan* evolved and the capacity of NGOs grew to deliver core activities, NGOs extended their services from prioritising the female sex worker programme to targeting high risk men also. Moreover, some activities such as STI service provision and community mobilisation were added to the core package of peer education. For example, community mobilisation started with relatively modest activities such as the provision of drop-in centres, but as more members of key populations were involved activities widened and intensified [21]. Syphilis testing was also difficult to implement initially because of quality assurance for tests but was instituted later, as was verbal screening for tuberculosis (although it is not commonly considered part of an HIV prevention intervention). For those countries planning services in the future, budgeting should therefore reflect an evolving change of scope over time.

We did not find a decrease in the variation of unit costs at the service level as scale-up progressed. This finding is somewhat surprising as learning effects are important to consider when examining the efficiency in the rapid scaling-up of HIV prevention activities [13], particularly when knowledge is transferred through the support activities of SLPs. While the computerised management information system took a year or so to establish, NGO activities were carefully monitored and progress fed back to them through the period. In principle these learning effects should lead to more standardised approaches and uniform costs. Moreover, the variation in unit cost should also have been minimised, as NGOs who did not comply with the financial procedures were dropped by a programme and replaced with new NGOs. Our findings therefore suggest that either the budgeting process was not sufficient attuned to promote efficiency, or that as the programme evolved the scope of services funded became increasingly heterogeneous across NGOs. Further work is being undertaken using econometric methods to better understand the main determinants of this cost variation.

When interpreting our results, care should be taken to consider the quality of our data. This study is the largest study to date of HIV prevention costs, it is also is the only study that collects data over time. However, conducting a study at this scale, using primarily top down methods, means that the quality of any disaggregated findings by activity may be less robust than studies that employ more site intensive methods, such as comprehensive time in motion studies. Moreover, although every effort was made to include donated goods, it is likely some sites' data was under-reported (although where it was closely monitored the values were a very low proportion of total costs (<5%)). Another challenge also is the allocation of above service level costs and indirect costs generally. While our approached is based on interviews and detailed analysis of expenditure reports, due to the scale of the study we were not able to provide timesheets to all above service level staff. The most important limitation of the data however is on the output side and the use of routine data to measure the level of service utilisation. Programme indicators in the initial years lacked consistency across the states and different NGOs may have started reporting at different times. This may have impacted the estimation of unit costs in the first year of the programme, resulting in an over-estimation of unit costs in the first year. Moreover, routine data may be subject to various biases, including an incentive to demonstrate strong performance by NGO level managers.

Finally, during our study we learned several important practical and methodological lessons. Firstly, we were able to conduct our study within a reasonable cost, due to our reliance on the top down method; and our complete access to all expenditure data, CMIS data, programme staff and key resource data. While this approach is not always feasible or desirable, the extensive time taken and effort made at the beginning and throughout the study by local staff to involve participants was pivotal to the ability of the costing team the data required. Both the comprehensiveness and longitudinal nature of the dataset provide the potential for econometric analysis of cost determinants, and provide an evidence base for those interested in resource allocation across interventions at varying scales. Such extensive estimates also help validate previous estimates made in small scale pilot settings. However, pragmatically, such large scale costing studies may be considered expensive; and thus may be most appropriate to questions focused on technical efficiency; and early pragmatic trials of the initial roll-out of new technologies and interventions. Even on these occasions it may be possible to take a more limited sampling approach and frequency of data collection. As part of our further econometric analysis of these results, we will explore whether collecting less cost data would substantially alter our policy recommendations; in order to inform and guide investment in HIV prevention costing methods going forward.

## Conclusion

This descriptive analysis of the costs of HIV prevention confirms that total costs, cost profiles and unit costs all evolve over time during the process of scale-up. In particular, policy makers and planners should note that above service costs can be considerable, that unit costs per person reached fall with scale, and that cost profiles by activity can change substantially over time. Further work exploring the optimal service package, how to reduce cost variation over time, and the efficiency of different models of above service support are recommended to ensure that other programmes learn fully from the *Avahan* experience and are able to achieve value for money.

## Supporting Information

Figure S1 **NGO economic cost by district and by activity 2004 to 2008 (US$ 2011).**
(TIF)

Figure S2 **SLP and NGO economic cost by district by input 2004 to 2008 (US$ 2011).**
(TIF)

Table S1 **Description of the activity considered at different organisational levels.**
(DOCX)

Table S2 **Costs considered for input categories.**
(DOCX)
