# Supplementary figures and images for: The Costs of Scaling Up HIV Prevention for High Risk Groups: Lessons Learned from the Avahan Programme in India
Source: PLoS One. 2014 Sep 9;9(9):e106582. doi: 10.1371/journal.pone.0106582 (PMC4159262; doi:10.1371/journal.pone.0106582)

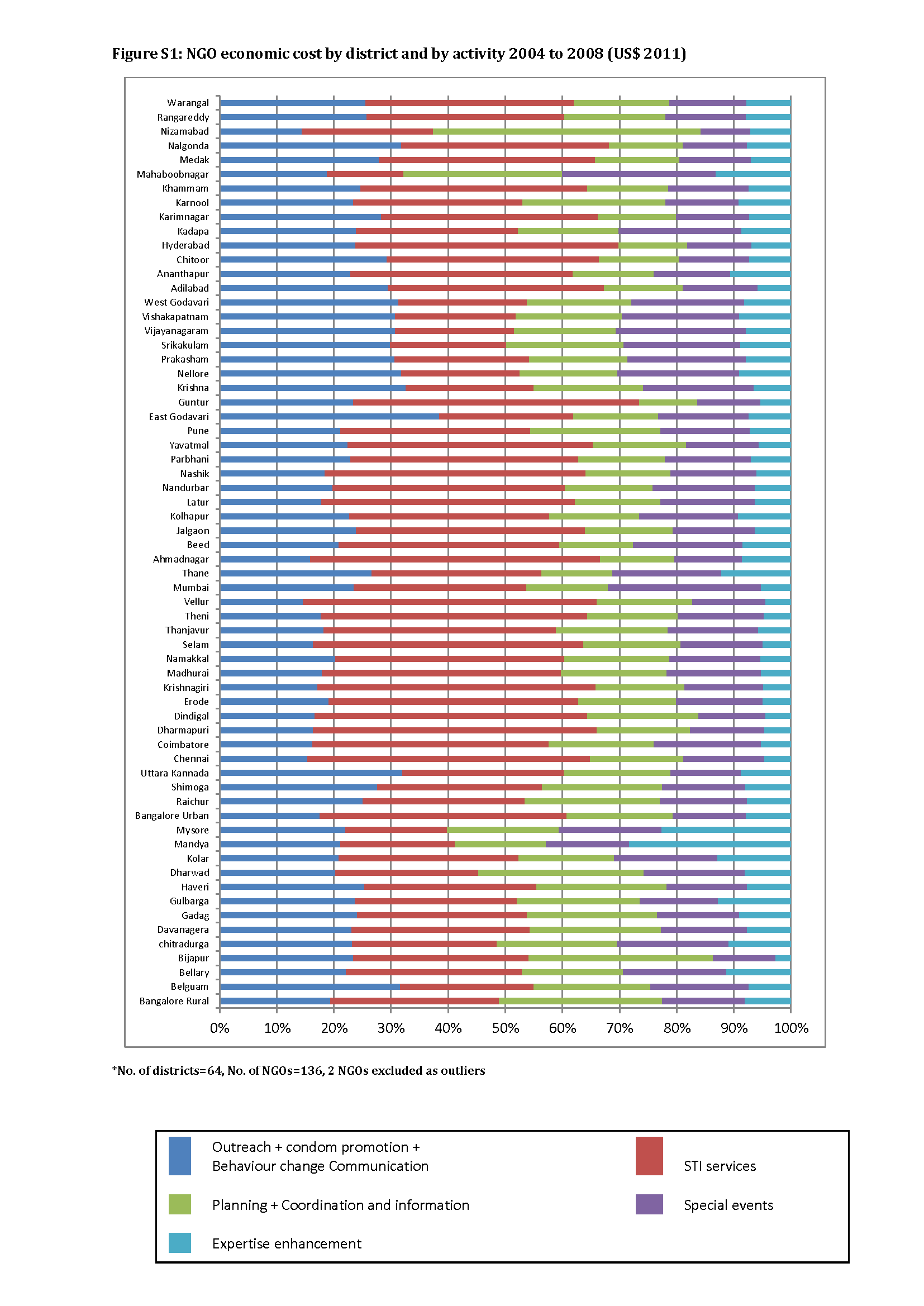

Supplement: Figure S1 — NGO economic cost by district and by activity 2004 to 2008 (US$ 2011). (TIF) [file pone.0106582.s001.tif]

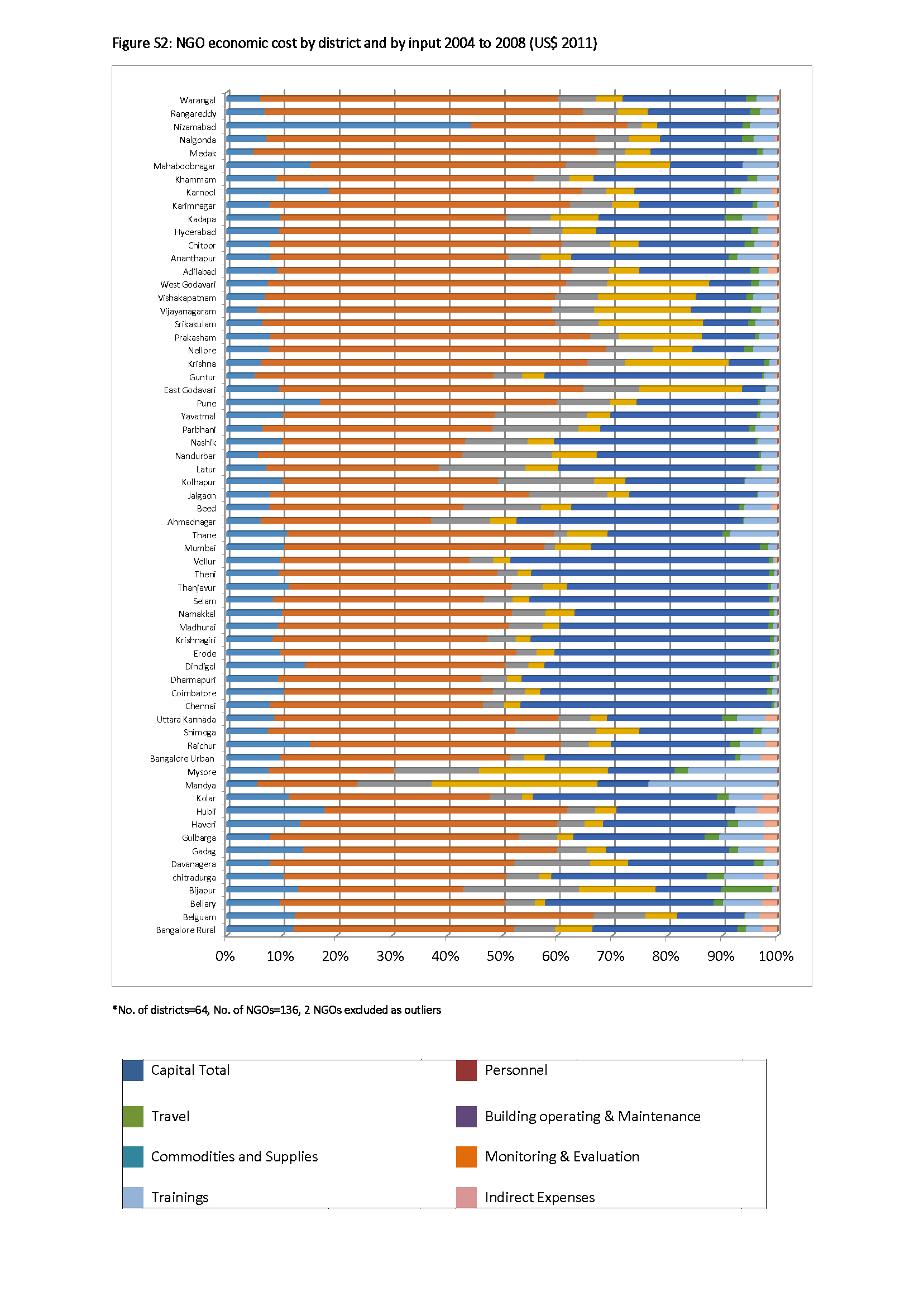

Supplement: Figure S2 — SLP and NGO economic cost by district by input 2004 to 2008 (US$ 2011). (TIF) [file pone.0106582.s002.tif]
