# Supplementary material for: The Costs of Scaling Up HIV Prevention for High Risk Groups: Lessons Learned from the Avahan Programme in India
Source: PLoS One. 2014 Sep 9;9(9):e106582. doi: 10.1371/journal.pone.0106582 (PMC4159262; doi:10.1371/journal.pone.0106582)
Supplement: Table S1 — Description of the activity considered at different organisational levels. (DOCX) [file pone.0106582.s003.docx]

**Supplementary Table S1: Description of the activity considered at different organisational levels**

| **Activity** | Description* |
| --- | --- |
| **Programme Level** |  |
| Support to media advocacy | All activities and related costs to assist positive media representation of the issues of high risk groups and to create an enabling environment to reduce stigma and wider acceptance of HIV prevention programmes for eg: through Public Service Announcements and embedded messaging in mainstream television programming, training journalists in the states, tracking stories, and placing positive stories in the local media |
| Support to policy advocacy | This included activities to sensitisize the police to reduce harassment of sex workers |
| Support to advocacy with societal leaders | These activities were for reducing the barries for uptake of programme services by high risk groups by advocating with local government leaders and at state level, improving resource allocation and also involving business sectors to support some activities |
| Support STI services | To maintain quality and ensure standardisation of services by regular monitoring, development of guidelines for STI syndromic management and clinic operation. Conducting operational research studies and also tracking the uptake of services |
| Support to community mobilization | Activities like vision building, learning site demonstration projects for increasing community participation and engagement in programmes by the key populations appropriate for local conditions. |
| Support to inter-personal communication | Support to develop interpersonal communication methodologies to promote behaviour change skills for field staff in communicating with key populations |
| Support to strengthening HIV positive networks | Raising capacity of PLHIV and supporting their networks. Especially for demand generation and also as a resource persons for training and sharing their experiences. |
| Support to programme management of expertise enhancement partner | Allocated expenses for Pan-Avahan capacity building partners who were provided grants to support specific technical areas for the state lead partners |
| Programme management general | General programme management activities of national office |
| **State Level lead implementing**  **Partner** |  |
| Expertise enhancement including technical assistance and training | Includes induction training and re-orientation for staff, workshops/trainings on specific topics of project management, immersion visits and field visits for staff |
| Information Education and communication | Development and printing of educationalmaterials in English and local languages. Creating modules on specific topics. Improve awareness through mass media, wall writings, streetplays and celebration of days like World AIDS day |
| Programme management | General activities for grant management including office set up, recruitment, indirect expenses and maintenance costs |
| Management information | Mapping, validation and baseline studies . Setting up and maintenance of centralised management information data. Support to surveys and special studies |
| Community mobilisation and enabling environment | Activities relate to establishment of safe spaces like Drop-in- centres, conduct of events for increasing key population participation, setting up of self help groups, supporting access to social entitlements, development of Community based organisation to increase ownership. Support to local advocacy to police, lawyers, local leaders, brothel madams and pimps. Supporting Peer convention and rallies. Setting up crisis response systems |
| Support and supervision | Includes staff support in the form of regional managers or STI managers to oversee the programme implementations of the NGOs/CBOs. Set up procurement mechanisms for consumables like condoms and STI drugs. Link with state AIDS control societies and collaborate with other organisations working in their areas. Plan NGO level and partner meetings to share learnings. |
| **Service Level (NGO)** |  |
| Outreach, condom promotion, behaviour change and communication | Includes peer outreach to provide programme servcies information and education on safe sexual practices, distribution of condoms and supplies. |
| STI Services | Referral to project STI services or referral doctors for STI treatment and follow-up. General medicines are also provided and referral for HIV counselling and testing. Syphills screening and treatment |
| Programme management | General activities like office set-up and maintenance, recruitment and project management related activities of NGO staff. Co-ordination with state lead partner .Reviews and meetings with field staff |
| Community mobilisation and enabling environment | Conduct of DIC events, advocacy with local leaders, celebration of important days and festivals. Running crisis response systems. Setting up of self help groups and participation in programme committes and active engagement to form community based organistion |
| Expertise enhancement including technical assistance and training | Trainings for peers, field level staff on programme implementation and workshops on skill development of project staff. |

* Costs considered for the activity include specific recurrent costs (like travel, consumables) and personnel, capital and general costs allocated for the activity
