# Supplementary material for: The Costs of Scaling Up HIV Prevention for High Risk Groups: Lessons Learned from the Avahan Programme in India
Source: PLoS One. 2014 Sep 9;9(9):e106582. doi: 10.1371/journal.pone.0106582 (PMC4159262; doi:10.1371/journal.pone.0106582)
Supplement: Table S2 — Costs considered for input categories. (DOCX) [file pone.0106582.s004.docx]

Supplementary Table S2: Costs considered for input categories

| **INPUT** | Costs considered (indicative list only and may include other items depending on the NGO) |
| --- | --- |
| Personnel | Salaries of all categories of staff, consultants and resource persons |
| Commodities and supplies | Costs of drugs, condoms, IEC materials |
| Training | Costs of space, experts, travel, food and accomodation for participants |
| Capital cost | Rental deposits, equipment, furniture, vehicle costs |
|  |  |
| Building operating & maintenance | Office supplies, electricity, water, repairs and maintenance |
| Travel | Fuel, vehicle hiring tickets, accomodation expenses and perdiems |
| Monitoring & Evaluation | Mapping and setting up of the management information systems, conducting routine surveys and reviews |
| Indirect Expenses | Reported overhead expenses |
